# Effect of Co-Existing Cations and Anions on the Adsorption of Antibiotics on Iron-Containing Minerals

**DOI:** 10.3390/molecules27228037

**Published:** 2022-11-19

**Authors:** Xiaoyu Guan, Juntao Guo, Hui Zhang, Shiyong Tao, Gilles Mailhot, Feng Wu, Jing Xu

**Affiliations:** 1 State Key Laboratory of Water Resources and Hydropower Engineering Science, Wuhan University, Wuhan 430072, China; 2 Hubei Key Laboratory of Water System Science for Sponge City Construction, Wuhan University, Wuhan 430072, China; 3 Hubei Key Lab of Biomass Resource Chemistry and Environmental Biotechnology, School of Resources and Environmental Science, Wuhan University, Wuhan 430079, China; 4 Universite Clermont Auvergne, CNRS, SIGMA Clermont, Institut de Chimie de Clermont-Ferrand, F-63000 Clermont-Ferrand, France

**Keywords:** antibiotics, iron-containing minerals, adsorption, co-existing ions, natural surface water

## Abstract

The adsorption of antibiotics on minerals is an important process in their environment behavior. The adsorption behavior of antibiotics on iron-containing minerals and the effect of co-existing cations and anions were studied in this work. Magnetite, hematite, goethite and kaolin were selected as the representative minerals and characterized by SEM, XRD and BET. A total of eight antibiotics, including three quinolones, three sulfonamides and two mycins were chosen as the research targets. Results showed a higher adsorption amount of quinolones than that of sulfonamides and mycins on the surface of iron-containing minerals in most mineral systems. The adsorption isotherms of quinolones can be well fitted using the Freundlich models. The effects of five cations and five anions on the adsorption of quinolones were investigated, among which Mg^2+^, Ca^2+^, HCO_3_^−^ and H_2_PO_4_^−^ mainly showed significant inhibition on the adsorption, while the effects of K^+^, Na^+^, NH_4_^+^, Cl^−^, NO_3_^−^ and SO_4_^2−^ showed less. Natural surface water samples were also collected and used as media to investigate the adsorption behavior of quinolones on iron-containing minerals. The buffering capacity of the natural water kept the reaction solution at circumneutral conditions, and the adsorption amount was mostly promoted in the goethite system (from 0.56~0.78 μmol/g to 0.52~1.43 μmol/g), but was inhibited in the other systems (magnetite: from 1.13~1.33 μmol/g to 0.45~0.76 μmol/g; hematite: from 0.52~0.65 μmol/g to 0.02~0.18 μmol/g; kaolin: from 1.98~1.99 μmol/g to 0.90~1.40 μmol/g). The results in this work help to further understand the transportation and fate of antibiotics in an aqueous environment.

## 1. Introduction

The environmental behavior of pharmaceutical and personal care products (PPCPs) has become a research hotspot in recent years. Antibiotics, as a PPCP, have also attracted much attention [1,2,3]. Antibiotics are widely used in medical treatments, agriculture, and animal husbandry, etc. due to their low price and efficient bactericidal effects, which leads to their release in the original form or metabolized form through multiple ways [4,5,6] At present, pharmaceutical pollutants are difficult to be effectively degraded by the traditional processes used in sewage treatment plants [7]. As a consequence of their extensive and frequent use, and the insufficient treatment processes, antibiotics have been detected in different environmental media in dozens of countries, including surface water, groundwater, soils and sediments [8,9,10]. It has been reported that the concentrations of antibiotics are up to μg/L level in surface water or wastewater [10]. Their accumulation in the environment will influence the ecosystem balance, induce the growth of resistant bacteria, and pose an adverse effect on human health [11,12]. Therefore, it is of great significance to investigate their environmental behavior.

Iron-containing minerals, including iron (hydr)oxides and clay minerals, are widely distributed in nature. The existence of these iron-containing minerals affects the environmental behavior of other substances co-existing in the environmental media, especially the mobility, transformation and fate of contaminants [13,14]. The selection and application of environmentally friendly, low-cost and high-efficiency materials for the treatment of environmental pollution is a hot research topic at present [15]. Iron-containing minerals have these characteristics mentioned above, and their utilization in water treatment has received much attention. Many studies have reported the adsorption behavior of antibiotics on mineral surfaces, which would facilitate the removal of these pollutants from water in both the natural water systems by self-purification and the waste water treatment systems by artificial measures. Roca Jalil et al. [16] studied the adsorption kinetics and isotherms of ciprofloxacin on montmorillonite, and considered that the solubility of antibiotics and environmental pH were important factors affecting the adsorption reaction. Guo et al. [17] investigated the effect of pH on the adsorption of sulfamethazine on goethite, considering that the π-π electron donor–acceptor interactions and outer- and inner-sphere complexions might be the dominating adsorption mechanisms under acidic and neutral conditions, while the adsorption in alkali conditions might be dominated by electrostatic interactions. Paul et al. [18] and Li et al. [19] also studied the adsorption mechanism of fluoroquinolones on iron-containing minerals, in which the influencing factors such as pH, temperature and co-existing ions were investigated.

Although many previous studies have reported the adsorption of antibiotics, most of them focused on the adsorption behavior of one antibiotic on one or several minerals or focused on the adsorption behavior of several antibiotics on the same mineral for comparison. When studying the influence of co-existing ions, the species of ions were also limited. As is widely known, natural surface water and sewage wastewater are rich in various anions and cations, and the presence of these ions may promote or inhibit the adsorption of pollutants on the particle surfaces due to various reasons. Therefore, it is significant to comprehensively consider the effects of these ions when describing the environmental behavior of antibiotics and evaluating their environmental risks. Based on this, this study selected a total of eight antibiotics as the research objects, including quinolones, sulfonamides and mycins, which are widely used in daily life and have been widely found in natural surface water and sediments. Their adsorption behaviors on magnetite (Mag), hematite (Hema), goethite (Goe) and kaolin (Kao) were compared. The effects of 10 ions, including five cations and five anions, on the adsorption behavior of antibiotics were investigated. The adsorption capacity of these pollutants in the actual environment was studied by using natural surface water as the media. The findings in this work will help with the understanding of the fate of antibiotics in complicated media.

## 2. Results and Discussion

### 2.1. Minerals Characterization

**Figure 1:**
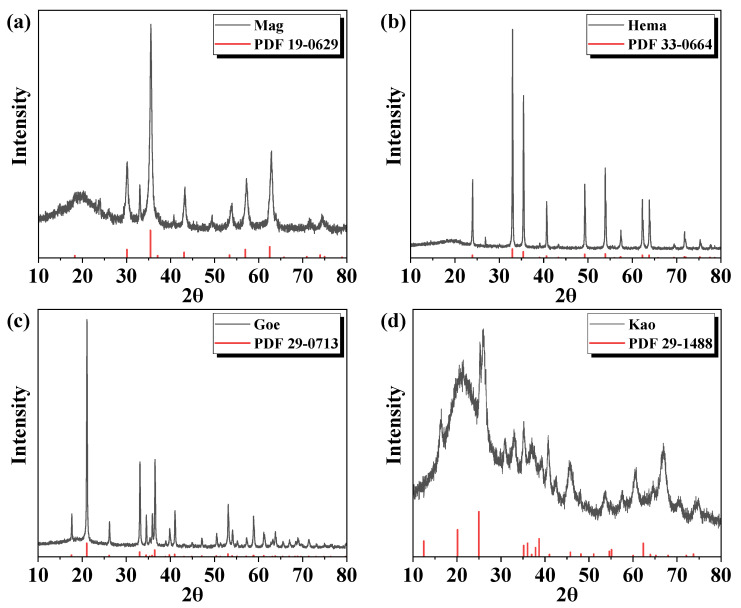
XRD patterns of (**a**) Mag, (**b**) Hema, (**c**) Goe, and (**d**) Kao.

The XRD patterns of each mineral particle ranging from 10° to 80° were presented in Figure 1a–d. The peaks of Mag, Hema, and Goe were all sharp and regular since these were iron oxides, and the peaks of Kao were less sharp since it was a clay mineral. For all the minerals, the 2 Theta values of the sharp diffraction peaks were consistent with the standard XRD cards.

**Figure 2:**
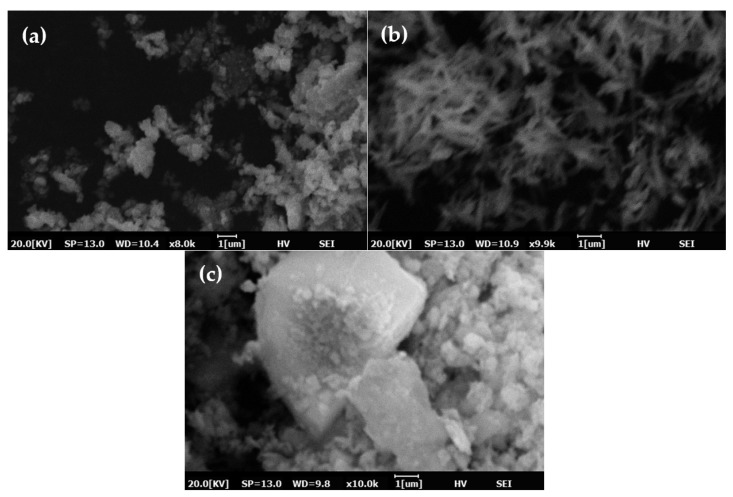
SEM images of (**a**) Hema, (**b**) Goe, and (**c**) Kao.

The morphology of three mineral particles is shown by SEM images in Figure 2a–c, and Mag was not performed due to the limitation of the used SEM equipment, which is not applied to magnetic materials. Hema was characterized by small particles with uniform size and shape; Goe was characterized by a fine needle shape with uniform size and shape; Kao was comprised of larger particles and exhibited non-uniform size and shape.

**Figure 3:**
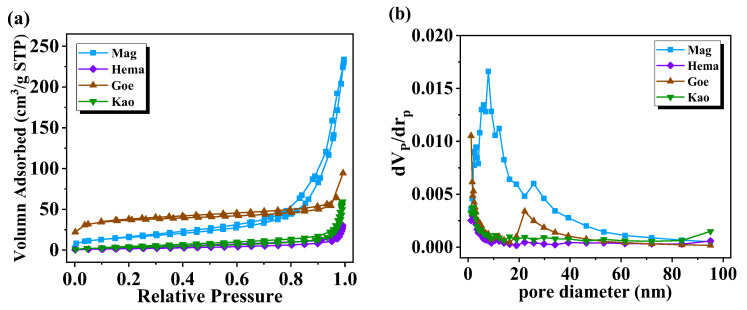
(**a**) N_2_ adsorption-desorption isotherms and (**b**) pore size distributions of Mag, Hema, Goe and Kao.

Table 1 presented the BET analysis results of each mineral particle, and Figure 3 presented the adsorption-desorption isotherms and pore size distributions. The pore volume of Kao was significantly larger than that of the other mineral particles, while its pore size was opposite. Kao also has the largest BET surface area among the four minerals. The BET surface area of the other three oxides was in the following order: Mag > Goe > Hema. The adsorption-desorption isotherm of Mag was similar to type II, indicating that there was almost no hysteresis phenomenon. The isotherms of Hema, Goe and Kao were type IV, which have hysteresis loops, indicating the existence of porous structure. The pore size distribution curves showed that the pore size of Mag mainly focused on 10 nm, and the co-existence of mesopores and micropores occurred in Mag and Geo. For Hema and Kao, the pore sizes were less than 2 nm.

### 2.2. Adsorption Isotherms

**Figure 4:**
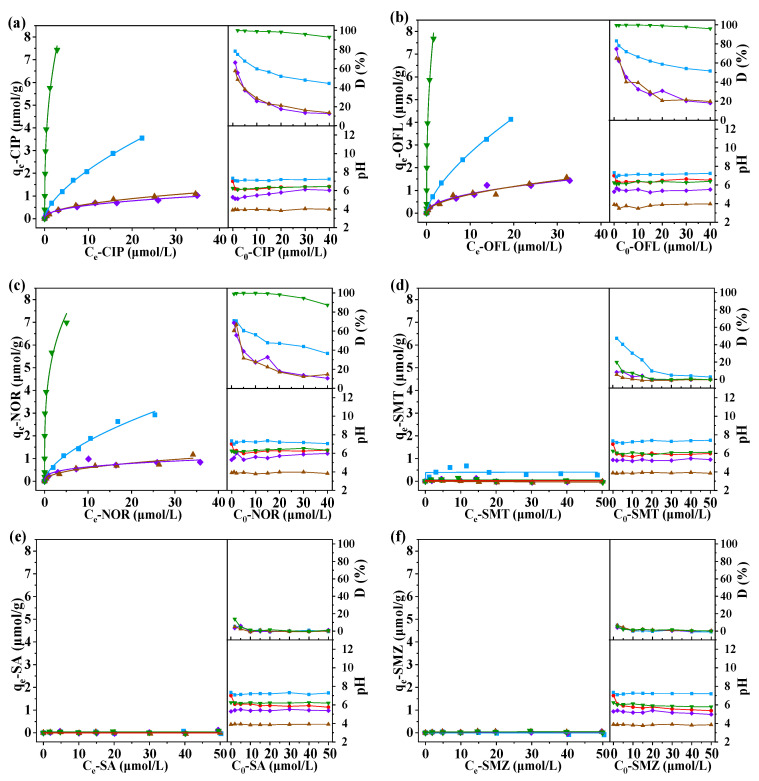

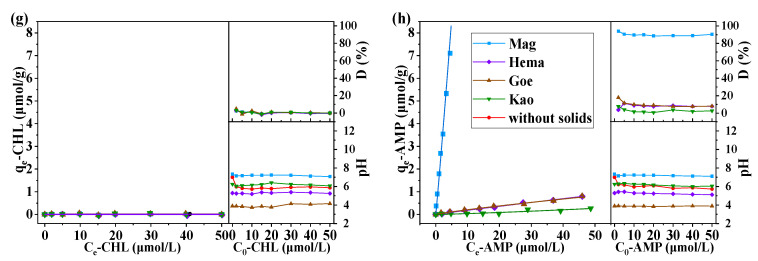
Adsorption of (**a**) CIP, (**b**) OFL, (**c**) NOR, (**d**) SMT, (**e**) SA, (**f**) SMZ, (**g**) CHL, and (**h**) AMP on iron-containing minerals. Conditions: dosage of solids = 5 g/L.

The adsorption behaviors of the selected eight antibiotics on the surface of four iron-containing minerals were investigated. Figure 4 showed their adsorption isotherms, adsorption percentages and the pH values of the solution after the reaction. Table 2 showed the calculated adsorption isotherm coefficients.

As can be seen from the results, all three of the investigated quinolones showed obvious adsorption with regard to the four minerals. The adsorption percentage ranged between 57.5~99.9% at low concentration conditions and decreased to 10.5~96.0% at high concentration conditions. The adsorption amount increased with the initial concentration of antibiotics, and the adsorption isotherms can be well fitted by Freundlich models (most of the R^2^ were >0.92). The obvious adsorption of the quinolones on solids has been widely reported in the previous studies [20,21]. In those works, the carboxyl groups and keto groups were considered as the key reason for the interaction between quinolones and solid surfaces. The three investigated sulfonamides only showed slight adsorption at low concentration conditions, and concentrations in the supernatant showed insignificant changes after the adsorption at high concentration conditions. The calculated adsorption amounts were very low under the varied initial concentration conditions; thus many adsorption isotherms cannot be fitted by Freundlich models. The weak adsorption ability of sulfonamides on solids has also been reported previously, which was similar to our experimental results [22,23]. This may be due to the lack of the functional groups in the sulfonamides that can be complexed with the iron on the minerals’ surface. The adsorption of the investigated mycins on the mineral surface was even weaker in the most investigated systems, except for the adsorption of AMP on Mag. AMP has a higher adsorption ability at circumneutral or weak basic conditions, as the deprotonated carboxyl groups of AMP in its zwitterionic form or anionic form contributed to its adsorption on the surface with a positive charge [24]. The insignificant adsorption results of CHL was consistent with the studies of Liu et al. [25] and Li et al. [26], which might be due to its lack of such a complexable group.

Among the investigated 4 iron minerals, Kao showed the best adsorption ability for quinolones, and the other followed the order of Mag > Hema ≈ Geo. For mycins (specifically AMP), the adsorption on Kao was not obvious, whereas the adsorption capacity of Mag was relatively strong. Mechanism studies have previously shown that a large number of surface hydroxyl groups formed by iron hydroxides contacting with water could react with the carboxyl groups of antibiotic molecules, resulting in adsorption [18,22]. In contrast, clay minerals interacted with antibiotics through their special layered structure, and adsorbed the solute through ion exchange [19,27]. These reasons may explain the adsorption mechanism of antibiotics on Mag, Hema, Goe and Kao. Besides, the electrostatic effect may also have a certain contribution, which was highly affected by the pH of solutions [28,29]. The pH values of the supernatants after the reaction were determined, and results were also given in Figure 4. The ending pH of the reaction solution was mainly affected by the properties of mineral particles, and the added antibiotics showed little effect due to the low concentration in this work and their weak dissociation ability in the aqueous solution. It can be found that the pHs of the supernatants from the systems of Mag, Hema, Goe and Kao were basically in the ranges of 6.8~7.5, 5.0~6.5, 3.5~4.2 and 5.8~6.7, respectively. Since pH is an important factor affecting the adsorption behavior, the adsorption capacity of different antibiotics on the surface of particles is not only related to the properties of the antibiotics and the surface of particles, but is also related to the pH of solution. Nevertheless, for the adsorption isotherm, the pH of the reaction solution was dominated by the minerals due to their large dosage, and the pHs were very close in the same suspension system.

According to the preliminary results, the adsorption of sulfonamides and mycins on the surface of selected iron-containing minerals were generally low, and large experimental errors may occur in the subsequent experiments. Quinolones, meanwhile, did not involve such problems. Therefore, only the three quinolones (CIP, OFL and NOR) were further used to investigate their adsorption on the surface of minerals under the influence of co-existing ions.

### 2.3. Effect of Co-Existing Ions on Adsorption

**Figure 5:**
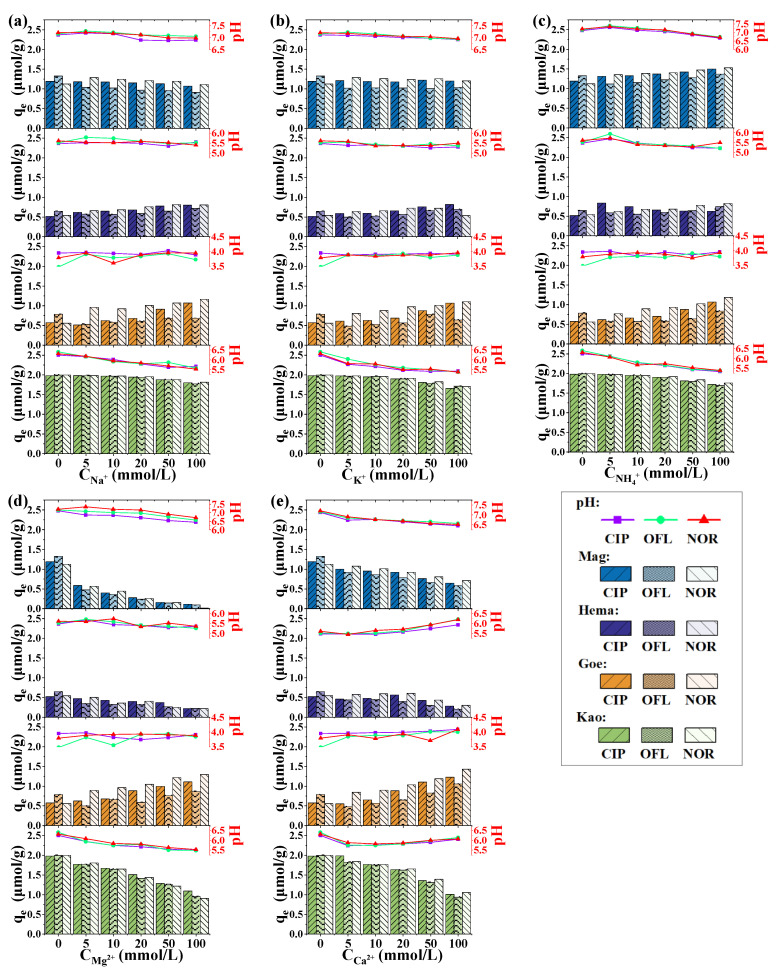
Effect of (**a**) Na^+^, (**b**) K^+^, (**c**) NH_4_^+^, (**d**) Mg^2+^, (**e**) Ca^2+^ on the adsorption of CIP, OFL and NOR. Bars represent the adsorption amount, and points represent the ending pH of the solution after the adsorption. Conditions: [CIP]_0_ = [OFL]_0_ = [NOR]_0_ = 10 μmol/L, dosage of solids = 5 g/L.

**Figure 6:**
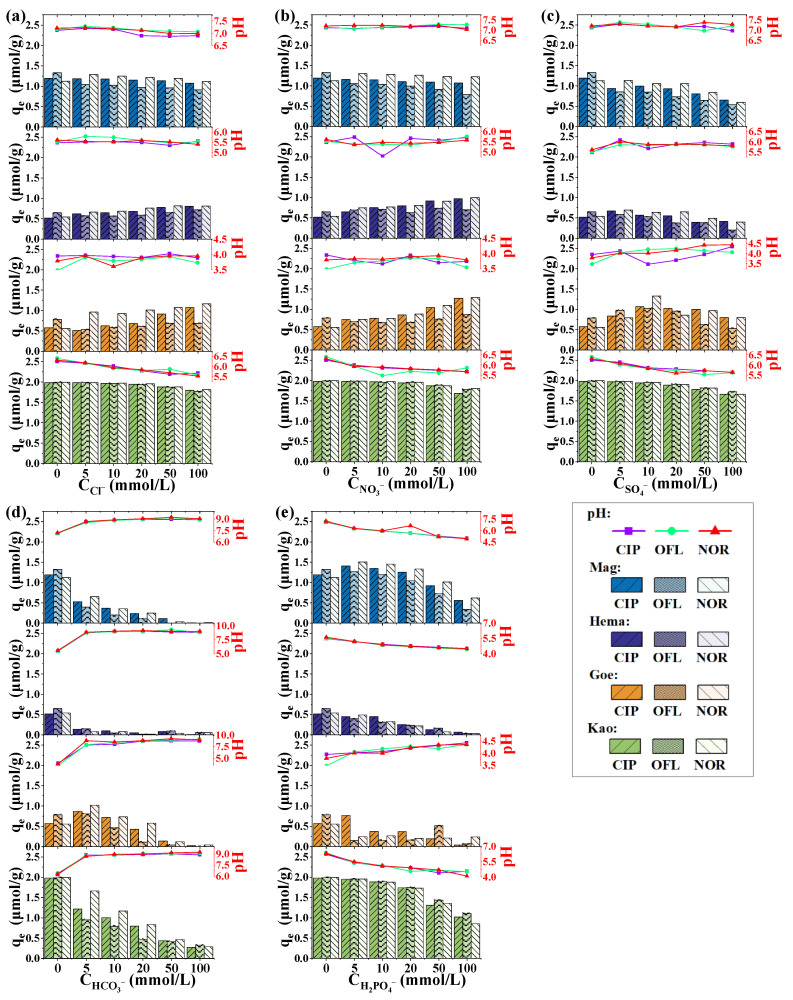
Effect of (**a**) Cl^−^, (**b**) NO_3_^−^, (**c**) SO_4_^2−^, (**d**) HCO_3_^−^, (**e**) H_2_PO_4_^−^ on the adsorption of CIP, OFL and NOR. Bars represent the adsorption amount, and points represent the ending pH of the solution after the adsorption. Conditions: [CIP]_0_ = [OFL]_0_ = [NOR]_0_ = 10 μmol/L, dosage of solids = 5 g/L.

The effects of both cations and anions on the adsorption of quinolones were investigated. When studying the influence of cations (Na^+^, K^+^, NH_4_^+^, Mg^2+^ and Ca^2+^), chlorine salts were consistently used; when studying the influence of anions (Cl^−^, NO_3_^−^, SO_4_^2−^, HCO_3_^−^ and H_2_PO_4_^−^), sodium salts were consistently used. The concentration range of each ion was 0–100 mmol/L. The adsorption amount of quinolones and the ending pH of supernatant after the adsorption were given in in Figure 5 and Figure 6.

The results showed that in the concentration range of 0–100 mmol/L, the five cations showed different effects of the adsorption of quinolones on iron-containing minerals. Among them, Na^+^, K^+^, and NH_4_^+^ showed only slight effects on the adsorption; Mg^2+^ and Ca^2+^ showed relatively significant effects, depending on the mineral systems. Specifically, in a Mag system, the presence of Na^+^, K^+^ and NH_4_^+^ showed insignificant effects on the adsorption of all three quinolones, while the presence of Mg^2+^ and Ca^2+^ significantly inhibited the adsorption. In a Hema system, the effects of cations were relatively weaker: the three monovalent cations led to a weak promotion on the adsorption, while the two divalent cations led to a weak inhibition. In a Goe system, all the five cations promoted the adsorption amount, and in a Kao system, all the cations reduced the adsorption amount at high ionic concentrations. Overall, the influence degrees of divalent cations were higher than monovalent cations.

The investigated anions also showed different effects on the adsorption in each mineral system. In a Mag system, Cl^−^ and NO_3_^−^ showed insignificant effects on the adsorption, while the other anions showed more or less inhibition. In both the Hema and Goe system, Cl^−^ and NO_3_^−^ slightly promoted the adsorption, and SO_4_^2−^ slightly inhibited the adsorption, while HCO_3_^−^ and H_2_PO_4_^−^ strongly inhibited the adsorption. In the Kao system, a slight inhibition can be observed in the presence of Cl^−^, NO_3_^−^ and SO_4_^2−^, and the inhibition was much stronger in the presence of HCO_3_^−^ and H_2_PO_4_^−^. It was noteworthy that the addition of Cl^−^, NO_3_^−^ and SO_4_^2−^ had little effect on the pH of the solution, while the introduction of HCO_3_^−^ and H_2_PO_4_^−^ led to a much more obvious change in pH due to their dissociation in water.

As has been pointed out, pH conditions may strongly affect the adsorption ability in various systems [16,20,30]. Comparing the effect of each ion in this work, it was found that the presence of K^+^, Na^+^, NH_4_^+^, Cl^−^, NO_3_^−^ and SO_4_^2−^ did not change the pH of the systems that much, while the presence of Mg^2+^, Ca^2+^, HCO_3_^−^, and H_2_PO_4_^−^ affected the pH of the systems more strongly. Therefore, the mechanisms of these 10 ions affecting the adsorption amount would be integrated, including the effect of ionic strength, the effect of competitive adsorption, the effect of complex formation, and the effect of pH change. The p*K*_a_s of the three quinolones investigated in this study were in the circumneutral range (6.30 and 8.61 for CIP, 6.20 and 8.55 for NOR, 6.13 and 8.21 for OFL) [31]. A higher adsorption amount was more likely to be achieved in the circumneutral range for these quinolones, given that extreme pH conditions (e.g., the presence of HCO_3_^−^ results in pH > 9, and the presence of H_2_PO_4_^−^ results in pH < 5) may exacerbate the electrostatic repulsion and weaken the hydrophobic interaction between the solute compounds and solid surface [32,33].

However, even in the systems with limited pH variation, the significant effects of some ions on the amount of adsorption can still be observed. It is generally recognized that the increase of ionic strength is not conducive to the out-sphere complexation, but does not affect or even promotes the inner-sphere complexation [34]. As reported before, both out-sphere complexation and inner-sphere complexation may contribute to the adsorption of quinolones, and the changes in the adsorption caused by the increase of ionic strength would depend on the varied contribution of the two complexation modes in each system [4,34,35]. Moreover, in the presence of co-existing cations, the formation of both the cationic bridges and cation-antibiotic soluble complexes may contribute to the changes of the adsorption amount. A cationic bridge refers to the role of metal ions acting as bridges to connect antibiotics and mineral particles to promote the adsorption behavior of antibiotics [36,37]. Cheng et al. [38] and Pei et al. [39] reported the bridging role of Cu^2+^ in quinolones adsorption by forming the metal-antibiotic complexes which have higher affinity than the antibiotics themselves. In contrast, Cheng et al. [38] also found that Mg^2+^ could complex with oxolinic acid, forming a more soluble specie, which resulted in the decrease of the adsorption of oxolinic acid at neutral conditions. Competitive adsorption, whereby the existing free ions (especially anions) in the solution compete for adsorption sites and thus suppress antibiotics adsorption, has also been widely reported [40,41]. Since the carboxyl group of quinolones is the important functional group for its adsorption, the bicarbonate, which has a similar structure, may also sorb on the solid surfaces, competing with quinolones.

The above mechanisms explain the effect of co-existing ions on the adsorption behavior, while the effect of the same ion in different suspended systems varied, indicating that the key mechanism of quinolones adsorption in each system is different, as well as the affecting mechanisms of ions.

### 2.4. Effect of Surface Water on Adsorption

**Figure 7:**
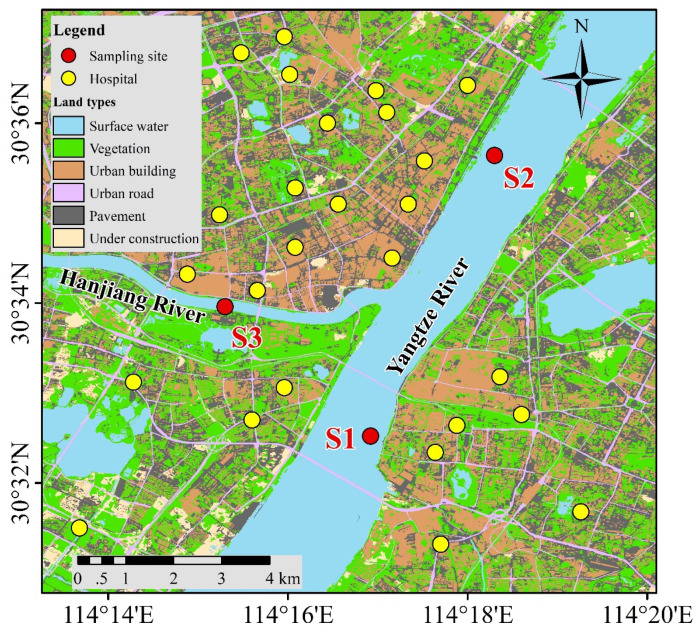
Map of sampling sites.

To assess the adsorption behavior in the media of natural surface water, two samples (S1 and S2) were collected from Yangtze River and one sample (S3) was collected from the Hanjiang River in Wuhan. The sampling sites are surrounded by dense residential areas with hospitals, as shown in Figure 7. The concentration of each ion specie in the collected surface water samples was analyzed, and the results were shown in Table 3. Most of the analyzed ions were at 10^−1^–10^1^ mmol/L level, except phosphate, which was below the detection limit, and ammonia, which was at 10^−3^ mmol/L level.

**Figure 8:**
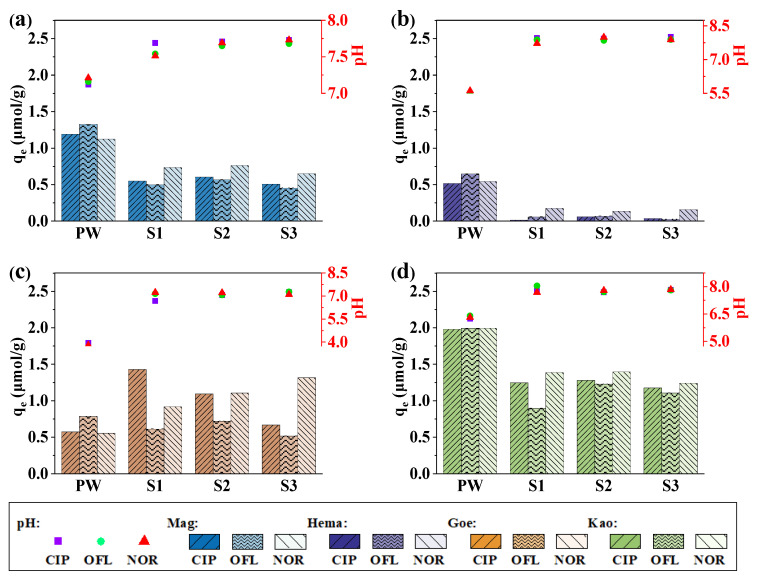
Adsorption of CIP, OFL and NOR on (**a**) Mag, (**b**) Hema, (**c**) Goe, and (**d**) Kao in the media of surface water. Bars represent the adsorption amount, and points represent the ending pH of the solution after the adsorption. Conditions: [CIP]_0_ = [OFL]_0_ = [NOR]_0_ = 10 μmol/L, dosage of solids = 5 g/L, PW means 10 mL ultrapure water, S1–S3 were mixtures of 2 mL antibiotic stock solution and 8 mL collected natural surface water.

Figure 8 shows the adsorption amount of the three quinolones on different minerals in the media of surface water, and the ending pHs of the solution after the reaction are also shown. The pH values after the adsorption reaction were steadily in the range of 7~8 in each mineral system due to the strong buffer effect caused by bicarbonate. The effect of the three surface water media on the adsorption behavior of quinolones was consistent, that is, the adsorption of antibiotics on the surfaces of Mag, Hema and Kao was significantly inhibited, and the adsorption on the surface of Goe was promoted. Combined with the discussion above, both the presence of Mg^2+^, Ca^2+^ and HCO_3_^−^ ions, and the changes of pH, played a major role in the changes of the adsorption amount. This indicates that in the environmental self-purification process, though antibiotics can be removed from the over-lying water through binding on the suspended particles in water, the removal efficiency can be inhibited by the co-existing ions, thus slowing down the self-purification rate.

## 3. Materials and Methods

### 3.1. Chemicals

Ciprofloxacin (CIP, C_17_H_18_FN_3_O_3_, purity > 98%), Ofloxacin (OFL, C_18_H_20_FN_3_O_4_, purity > 98%), Norfloxacin (NOR, C_17_H_20_FN_3_O_3_, purity > 98%), Sulfamethazine (SMT, C_12_H_14_N_4_O_2_S, purity > 99%), Sulfanilamide (SA, C_6_H_8_N_2_O_2_S, purity > 98%), Sulfamethoxazole (SMZ, C_10_H_11_N_3_O_3_S, purity > 98%), Chloramphenicol (CHL, C_11_H_12_Cl_2_N_2_O_5_, purity > 99%) and Ampicillin (AMP, C_16_H_19_N_3_O_4_S·H_2_O, purity > 96%) were purchased from Aladdin Industrial Co., Ltd. (Shanghai, China). Magnetite (Mag, γ-Fe_2_O_3_, purity > 98%) were purchased from Aladdin Industrial Co., Ltd. (Shanghai, China), Hematite (Hema, Fe_2_O_3_, 99.8%-Fe) were purchased from Macklin Biochemical Co., Ltd. (Shanghai, China), Goethite (Goe, FeO(OH), 30~60%Fe) were purchased from Sigma-Aldrich (St. Louis, MO, USA), and Kaolin (Kao, Al_2_Si_2_O_5_(OH)_4_, CP) were purchased from Sinopharm Chemical Reagent Co., Ltd. (Shanghai, China). All inorganic salts used in the experiment were analytically pure (AR) and purchased from Sinopharm Chemical Reagent Co., Ltd. (Shanghai, China). Formic acid (AR), Methanol (GR) and Acetonitrile (GR) were obtained from Sinopharm Chemical Reagent Co., Ltd. (China). Ultrapure water was produced by a water purification system (Ming-Che 24UV, Millipore, Burlington, MA, USA) and used for the solution preparation.

The stock solutions of antibiotics were prepared by dissolving the solid in ultrapure water without adding any other reagents. Therefore, the concentrations of antibiotics in the stock solutions were limited by their solubility. The stock solutions of inorganic salts (200 mmol/L) were also prepared by dissolving them in ultrapure water. All of the prepared stock solutions were kept out of the light.

### 3.2. Characterization of Minerals

The X-ray diffraction (XRD) patterns were recorded on a MiniFlex600 (Rigaku Corporation, Tokyo, Japan) using Cu−Kα radiation at a generator voltage of 30 kV and a tube current of 30 mA. The scanning electron microscopy (SEM) images were taken on an EM-30 Plus (COXEM, Daejeon Korea). The specific surface areas of samples were obtained on a Belsorp-mini II (MicrotracBEL Corporation, Osaka, Japan). The samples were degassed under vacuum at 250 °C for more than 5 h before measurement. The Brunauer–Emmet–Teller (BET) specific surface areas were calculated based on the linear part of the BET plot (*P*/*P*_0_ = 0.05–0.25). The Barrett–Joyner–Halenda (BJH) pore distributions were calculated based on adsorption branches, and total pore volumes were calculated based on the quantities of adsorbed nitrogen at the maximum relative pressure (*P*/*P*_0_ = 0.99).

### 3.3. Adsorption Experiments

Adsorption experiments were conducted in 15 mL polypropylene tubes. A 10 mL volume of solution containing the desired concentration of antibiotics was prepared in the tubes, and 0.05 g mineral particles were introduced into tubes. The suspensions were equilibrated with an up-to-end rotating mixer (MX-RL-Pro, Dragonlab, Beijing, China) at 70 r/min for 24 h under natural pH conditions, and then centrifuged at 7500 r/min by a centrifuge (TD5A, Cence, Changsha, China). The supernatants were withdrawn and the ending pH and antibiotic concentration of the solution after adsorption were further determined. The control groups were conducted following the same procedures above but without adding mineral particles.

For the experiments investigating the influence of co-existing ions, different volumes of the salt stock solution were also added to the reaction solution, making the reaction concentration to be 0, 5, 10, 20, 50, and 100 mmol/L, respectively. The remaining steps were the same as described above.

For the experiments investigating the adsorption of antibiotics in the surface water medium, 0.05 g mineral particles, 2 mL antibiotic stock solution and 8 mL collected surface water were mixed in the tubes to obtain the 10 mL reaction solution. The remaining steps were the same as described above.

### 3.4. Analysis Methods

The concentrations of antibiotics were determined using high-performance liquid chromatography (HPLC, LC-20, Shimadzu, Kyoto, Japan) with a C18 column (4.6 mm × 250 mm, 5 μm, Supelco Discovery, Sigma-Aldrich, St. Louis, MO, USA) and guarded by a 10 mm C18 guard column. The column was operated at 25 °C. The mobile phase, detecting wavelength and retention time for each antibiotic, are given in Table 4. The flow rate was 1.0 mL/min.

The contents of anions (Cl^−^, NO_3_^−^, SO_4_^2−^ and H_2_PO_4_^−^) and cations (Na^+^, K^+^, NH_4_^+^, Mg^2+^ and Ca^2+^) in collected surface water were determined by ion chromatograph (930 Compact, Metrohm, Herisau, Switzerland). The titration method was used to determinate the content of HCO_3_^−^.

### 3.5. Data Analysis

The adsorption equilibrium concentrations of each antibiotic were obtained by HPLC analysis, recorded as *C*_e_ (μmol/L). The adsorption percentage (D) can be calculated by the difference between the concentrations before and after the reaction according to equation 1, where *C*_0_ (μmol/L) represented the concentration before the adsorption reaction. The equilibrium adsorption amount on a per unit mass of solid (*q*_e_, μmol/g) were obtained according to equation 2, where *m* was the added amount of minerals (g) and *V* was the volume of the suspended reaction solution.

(1) $$D=\frac{C_{0}-C_{e}}{C_{0}}\times 100\%$$

(2) $$q_{e}=\frac{(C_{0}-C_{e})\times V}{m}$$

Adsorption isotherms were fitted using the Freundlich models (expressed as Equation (3)) due to the low investigated concentration of antibiotics limited by their solubility. *K_f_* and *n*, as the Freundlich adsorption constants, represented the adsorption capacity and adsorption intensity, respectively.

(3) $$q_{e}=K_{f}\cdot C_{e}{}^{\frac{1}{n}}$$

## 4. Conclusions

The adsorption of quinolones, sulfonamides and mycins on 4 kinds of iron-containing minerals were studied in this work. With the initial concentration of 10 μmol/L, the adsorption percentages of quinolones, i.e., CIP, OFL and NOR, ranged between 25.8~99.0%, 32.4~99.7% and 27.2~99.7% in different mineral systems, respectively, which were much higher than that of sulfonamides (SMT, SA, SMZ) and mycins (CFL, AMP) in almost all the mineral systems. Co-existing ions affected the adsorption behavior of quinolones, and varied with ions and mineral systems. Mg^2+^, Ca^2+^, HCO_3_^−^ and H_2_PO_4_^−^ mainly showed significant inhibition on the adsorption, which might be caused by the joint-effect of ionic strength, competitive adsorption, complex formation, and pH change. In the meantime, the effect of K^+^, Na^+^, NH_4_^+^, Cl^−^, NO_3_^−^ and SO_4_^2−^ were less significant than the former four ions. The adsorption of quinolones also occurred in the media of natural surface water, with higher adsorption amounts on the Goe surface (increased from 0.56~0.78 μmol/g to 0.52~1.43 μmol/g) and lower adsorption amounts on the other three mineral surfaces than the ultrapure water media. Investigating the transportation of contaminants under the influence of co-existing ions helps to further understand their geochemical processes in the aqueous environment.

## Figures and Tables

**Table 1:** BET analysis results of the iron-containing minerals.

Minerals	Total Pore Volume (cm^3^/g)	Average Pore Size (nm)	BET Surface Area (m^2^/g)
Mag	0.33	24.64	54.21
Hema	0.04	21.63	8.15
Goe	0.09	26.64	12.77
Kao	0.14	4.27	132.96

**Table 2:** Fitted parameters for different antibiotics adsorption isotherms.

Antibiotics	Mineral	K_f_	n	R^2^
CIP	Mag	0.50	1.58	0.999
	Hema	0.23	2.47	0.987
	Goe	0.23	2.20	0.993
	Kao	5.22	2.77	0.992
OFL	Mag	0.58	1.51	1.000
	Hema	0.30	2.19	0.961
	Goe	0.25	1.96	0.958
	Kao	6.65	2.65	0.969
NOR	Mag	0.45	1.67	0.988
	Hema	0.31	3.25	0.826
	Goe	0.21	2.26	0.922
	Kao	4.65	3.51	0.932
SMT	Mag	0.39	130.13	0.339
	Hema	/	/	0.022
	Goe	/	/	0.199
	Kao	/	/	0.097
SA	Mag	/	/	0.022
	Hema	/	/	0.014
	Goe	/	/	0.001
	Kao	/	/	0.121
SMZ	Mag	/	/	0.001
	Hema	0.02	12.98	0.146
	Goe	0.04	10.73	0.415
	Kao	0.02	5.38	0.194
CHL	Mag	/	/	0.034
	Hema	/	/	0.006
	Goe	0.01	5.03	0.056
	Kao	/	/	0.018
AMP	Mag	1.56	0.94	0.983
	Hema	0.02	1.06	0.994
	Goe	0.03	1.17	0.988
	Kao	0.00	0.81	0.800

Note: / in the table indicates that the fitting results are very poor and the data are meaningless.

**Table 3:** Concentrations of cations and anions in surface water samples (mmol/L).

Sample	Na^+^	K^+^	NH_4_^+^	Mg^2+^	Ca^2+^
S1	0.490	0.068	0.003	0.388	0.902
S2	0.589	0.071	0.003	0.358	0.927
S3	0.465	0.070	0.003	0.402	1.000
**Sample**	**Cl** **^−^**	**NO** **_3_** **^−^**	**SO** **_4_** **^2−^**	**H** **_2_** **PO_4_^−^**	**HCO** **_3_** **^−^**
S1	0.538	0.123	0.422	n.d.	2.746
S2	0.622	0.153	0.438	n.d.	2.480
S3	0.524	0.121	0.425	n.d.	2.593

Note: n.d. = not detected.

**Table 4:** HPLC detection conditions.

Antibiotics	Detecting Wavelength (nm)	Mobile Phase				Retention Time (min)
		Methanol	Formic Acid (5‰)	Ultrapure Water	Acetonitrile	
CIP	275	0	20%	62%	18%	5.4
OFL	287	0	20%	62%	18%	5.2
NOR	283	0	20%	62%	18%	5.1
SMT	266	40%	20%	40%	0	4.5
SA	259	10%	20%	70%	0	4.5
SMZ	266	40%	20%	40%	0	5.0
CHL	279	0	0	60%	40%	5.1
AMP	210	0	10%	75%	15%	4.4

## Data Availability

The data that support the findings of this study are available from the authors upon reasonable request.

## Sample Availability

The materials used in the study are commercially available and can be purchased from the relevant firms.
